# Metabolic remodeling in takotsubo syndrome

**DOI:** 10.3389/fcvm.2022.1060070

**Published:** 2022-11-24

**Authors:** Ti Wang, Ting Xiong, Yuxue Yang, Bangyun Zuo, Xiwei Chen, Daxin Wang

**Affiliations:** ^1^The Hospital Affiliated to Medical School of Yangzhou University (Taizhou People’s Hospital), Taizhou, Jiangsu, China; ^2^Department of Cardiology, The Second Xiangya Hospital, Central South University, Changsha, Hunan, China

**Keywords:** takotsubo syndrome, metabolic remodeling, cardiac metabolism, systemic metabolism, metabolic therapy

## Abstract

The heart requires a large and constant supply of energy that is mainly the result of an efficient metabolic machinery that converges on mitochondrial oxidative metabolism to maintain its continuous mechanical work. Perturbations in these metabolic processes may therefore affect energy generation and contractile function directly. Metabolism characteristics in takotsubo syndrome (TTS) reveals several metabolic alterations called metabolic remodeling, including the hyperactivity of sympathetic metabolism, derangements of substrate utilization, effector subcellular dysfunction and systemic metabolic disorders, ultimately contributing to the progression of the disease and the development of a persistent and long-term heart failure (HF) phenotype. In this review, we explore the current literature investigating the pathological metabolic alterations in TTS. Although the metabolic dysfunction in takotsubo hearts is initially recognized as a myocardial metabolic inflexibility, we suggest that the widespread alterations of systemic metabolism with complex interplay between the heart and peripheral tissues rather than just cardiometabolic disorders *per se* account for long-term maladaptive metabolic, functional and structural impairment under this condition. Therapeutic strategies with the recent evidence from small clinical and animal researches, especially for targeting substrate utilization and/or oxidative stress, might be promising tools to improve the outcome of patients with TTS beyond that achieved with traditional sympathetic inhibition and symptomatic therapies.

## Introduction

Takotsubo syndrome (TTS) is an acute heart failure (HF) syndrome in which left ventricular (LV) dysfunction is transient and reversible (<21 days) with a typical wall motion abnormality of the LV apex predominantly precipitated by sudden, unexpected emotional or physical stress (1). The clinical presentation is characterized by generally abrupt onset of precordial pain, dyspnea, electrocardiogram changes (ST-elevation, T wave changes) and elevation of serum cardiac biomarkers without the evidence of culprit coronary disease (2). Historically, the commonly accepted belief of TTS was a self-limiting condition due to the recovery of the LV dysfunction in the days or weeks after onset (3). More recently, it has become clear that survivors of TTS may experience long-term cardiac and non-cardiac health problems, especially with metabolic alterations in addition to cardiac structural and functional changes, and be in poor outcomes (4). This condition is associated mostly with cardiac energetic impairment, deranged substrate utilization, oxidative and nitrosative stress, which may even contribute to the chronic HF phenotype in takotsubo patients (5, 6). Thus, given growing recognition of metabolic dysfunction as a critical component of TTS pathophysiology and prognosis, we believe the metabolic research in elucidating molecular mechanisms and even translating these alterations into novel therapeutic strategies presents an important need. In this review, we focus primarily on metabolic remodeling in TTS and discuss treatment options and whether targeting cardiac metabolic derangements might ameliorate the progression of disease and improve the ultimate consequences of cardiac structure and function.

## Epidemiology

With the greater awareness and detection of TTS, the true incidence of this condition has been increasingly recognized. At present, it is estimated that ≈2–3% of all patients and 5–6% of female patients for a suspected acute coronary syndrome have TTS (1, 3). Although it may be underdiagnosed and underestimated especially in patients coexisting with acute coronary diseases, the incidence of TTS is calculated to be ≈100 new cases per 1 million population per annum. Whereas TTS can occur in any age group, about 90% of patients are postmenopausal women with a mean age of 67–70 years and around 80% are older than 50 years. Women after the age of 55 years have a 10-fold greater risk of developing TTS than men (2, 4). The precise reason underlying this large sex disparity is unknown, but in recent years several studies suggest males with TTS least likely to get TTS may represent a particularly vulnerable population with a higher prevalence of cardiovascular complications and death in the acute period compared with their female counterparts. Larger studies are need to shed light on possible pathophysiologic mechanisms of this disorder.

## Metabolic flexibility of the normal heart

The healthy heart requires a large and constant supply of energy to sustain muscle contraction and ionic homeostasis due to a low ability for the heart to reserve energy (7). To fulfill the substantial energy requirement, ∼95% of the cardiac energy demand is a result of an efficient metabolic machinery that converges on mitochondrial oxidative metabolism (8). Most of this energy production in the form of adenosine triphosphate (ATP) is obtained by metabolizing a number of different energy substrates, including fatty acid (FA), glucose and lactate, ketones, and amino acids (9). Under normal conditions, the ∼70–90% of ATP produced is derived from FA oxidation, whereas the remaining ∼10–30% of ATP from the oxidation of glucose and lactate, as well as a minor amount of ketone bodies and certain amino acids (10, 11). However, the healthy adult heart is able to adapt its energy substrate use depending on the workload of the heart, energy substrate availability, and hormonal and nutritional state. This ability called metabolic flexibility allows the normal heart to shift between different substrates to ensure the necessary and continuous output of ATP in a wide variety of conditions (12). For instance, high rates of FA oxidation in the heart suppress glucose utilization, and vice versa, by a feedback mechanism known as the Randle cycle. Furthermore, the catalytic activity of rate-limiting enzymes, and transcriptional and translational processes are also involved in modulating the flux of substrates through oxidative pathways, such as inducing phosphorylation or acetylation of rate-limiting enzymes, association or dissociation with regulatory proteins, allosteric effectors (11, 13).

## Changes in cardiac sympathetic metabolism in takotsubo syndrome

**FIGURE 1:**
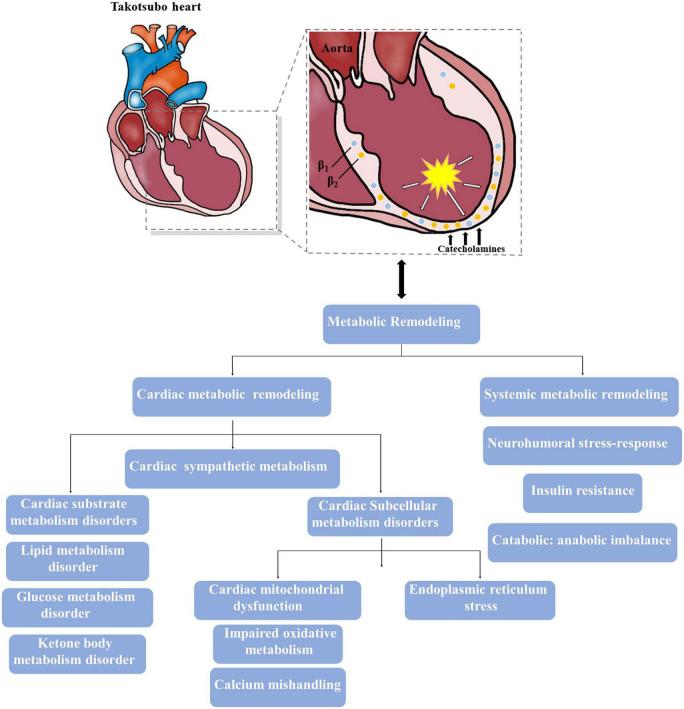
Major metabolic impairments involved in the takotsubo heart. The intense activation of catecholamine results in both cardiac and systemic metabolic remodeling, and theses altered metabolism also contribute to the progress of takotsubo syndrome (TTS).

Various groups favor the concept that the surge of catecholamine concentration might have a direct toxic effect on the ventricular myocardium in the acute phase of TTS (Figure 1). Such elevated catecholamines presented to cardiac adrenergic receptors may originate from circulating norepinephrine and epinephrine coupled with norepinephrine derived locally from sympathetic nerve terminals (14). Indeed, catecholamines released directly into the heart from nerve terminals are probably more toxic than those via the bloodstream (14). Previous evidence has already shown that norepinephrine spillover from sympathetic nerve terminals might induce myocardial injury, leading to contraction band necrosis, together with an increased extracellular matrix that contributes to cardiac structural and functional disorders during TTS (15). Moreover, excessive norepinephrine stimulation could further drive multivessel vasospasm mediated by α-adrenoceptors in the vasculature (16). In several myocardial biopsies of TTS patients, inflammatory cells infiltration, characteristic contraction bands and local fibrotic response have also been seen in the LV myocardium (17). However, it is interesting to note that the affected myocardium seems to has a high potential of structural reconstitution, which is in line with the transient wall motion abnormalities in most patients and suggests that an underlying protective mechanism is likely to limit acute myocardial injury in the presence of catecholamine storm. Paur et al. elegantly demonstrate this mechanism, showing that an excessive epinephrine level causes direct myocyte cardiodepression and cardioprotection through β_2_-adrenoceptor-activated G_i_ signaling (18). It is well established that both epinephrine and norepinephrine mediate positive inotropic responses through the activation of β-adrenoceptors G_s_ pathways, but an excessive epinephrine level triggers β_2_-adrenoceptor to switch from stimulatory G_s_ signaling pathways to inhibitory G_i_ pathways in the context of extreme stress that causes a negative inotropic response within the cardiomyocyte followed by apical ballooning (14, 19). The switch from G_s_ to G_i_ coupling may be a mechanism to confer protection against subsequent acute myocardial injury and preserve energy metabolites, whereas β_2_-adrenoceptor activation of the G_*i*_ pathway either switches back to G_s_ pathway or is internalized and degraded after the clearance of epinephrine surge, thus facilitating recovery of inotropic function (14, 20). In addition, a reverse apical-basal distribution of β_2_-adrenoceptors compared to the apical-basal gradients of norepinephrine β_1_-adrenoceptors and sympathetic nerve endings, which shows a higher density of β_2_-adrenoceptors in the apical than basal myocardium of the LV based on the experimental data of non-human mammalian hearts, might make the apex most sensitive to exaggerated catecholamine stimulation (19). Yet, current studies still lack detailed human evidences whether similarities of apical-basal gradients of β-adrenoceptors exist in human hearts. Further elucidating the dynamic information of β-receptor distribution with positron emission tomography (PET)-radiolabeled specific receptor ligands may help to broaden the understanding of pathophysiology in TTS.

## Changes in cardiac substrate metabolism in takotsubo syndrome

### Lipid metabolism disorder

**FIGURE 2:**
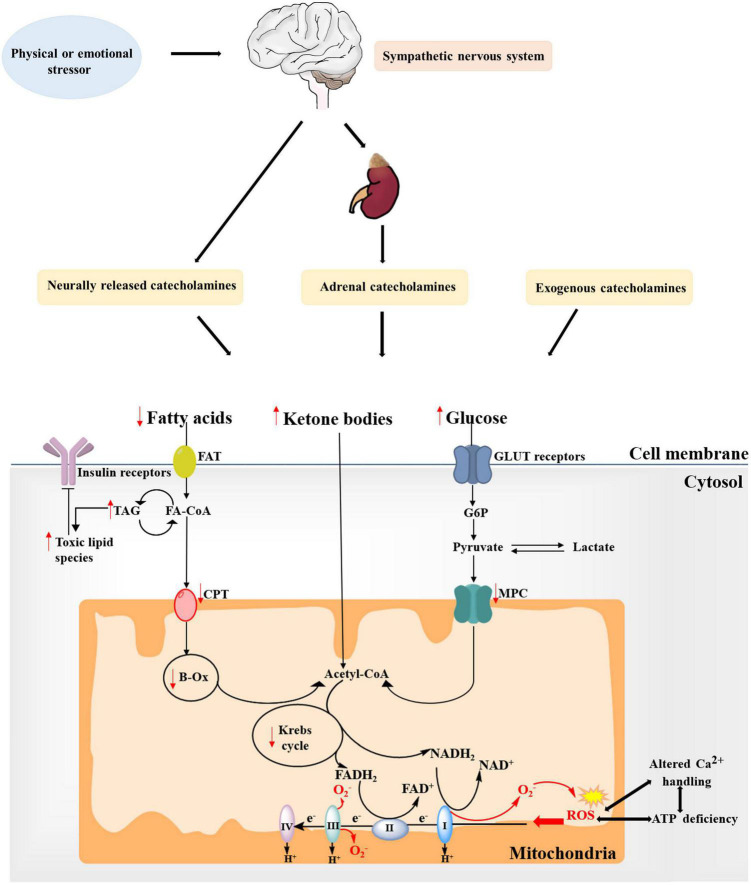
Alterations in substrate metabolism and mitochondria in takotsubo syndrome (TTS). Fatty acid uptake is impaired during the acute phase of catecholamine overstimulation, resulting in the accumulation of toxic lipid intermediates. Moreover, the concomitant decrease in overall glucose utilization further leads to reduced ATP production. Ketone bodies might become a more relevant source of energy in the takotsubo myocardium. These changes is also in-keeping with mitochondrial metabolism dysfunction, showing increased generation of mitochondrial reactive oxygen species (ROS) due to changes between the tricarboxylic acid (TCA) cycle and the electron transport chain (ETC) system, which may further increase ROS production and impair ATP production and Ca2+ handling to create a vicious cycle. Red arrows indicate changes in TTS. FAT, fatty acid transporter; FA-CoA, fatty acyl-CoA ester; TAG, triacylglycerol; CPT, carnitine palmitoyltransferase; β-Ox, β-oxidation; CoA, coenzyme A; G6P, glucose 6-phosphate; MPC, mitochondrial pyruvate carrier; GLUT, glucose transporter.

Most studies have revealed the effects of adrenergic activation on lipid metabolism in the myocardium, which could accelerate lipid uptake and lipid oxidation (14, 21). Whereas animal models provide evidence that intracellular lipid droplets significantly accumulate in cardiomyocytes during the acute phase of catecholamine overstimulation, which cause myocardial dysfunction in TTS, this metabolic shift is consistent with impaired myocardial FA metabolism, increased intramyocardial lipid accumulation and resultant fatty infiltration in endomyocardial or postmortem biopsy of hearts from patients with TTS (22, 23). The lipid over storage might be explained at least in part by the activation of the Poly(ADP-ribose) polymerase-1 (PARP-1) via SIRT-1 mechanism based on the observation of Bai et al. (24), which decreases energy production, inhibits relevant lipid export and downregulates FA oxidative metabolism. Of note, this toxic intramyocardial lipid accumulation is accompanied by a concomitant impairment in FA uptake (Figure 2). Imaging by PET and single photon emission computed tomography provide support to the decreased ability of apical akinetic areas of the heart to take up FA, as well as the reduction of long-chain FA uptake 3–5 days in post-takotsubo patients (25). These changes is also in-keeping with a shutdown of mitochondrial metabolism, showing a reduction of FA consumption, over storage of uncoupling of oxidative phosphorylation and consequent decline in ATP production, and lead to formation of reactive oxygen species (ROS) by creating lipotoxic intermediates and further aggravate cardiac dysfunction (22, 26, 27). Moreover, excessive lipid and its intermediates, serve as substrates for lipid peroxidation, perturb ion transport processes by impairing intracellular proteins and disrupting the integrity of biomembranes (28). It is reported that malignant arrhythmias in patients with TTS may partly originate from lipid overload mediated the disturbances of voltage-gated potassium ion currents and calcium ion (Ca^2+^) balance and prolongation of action potentials (22, 29). These lines of evidence suggest that oversupply of lipid-related substrates play an important role in the progression of TTS, and, therefore, improvements in cardiac lipid metabolism has been proposed to be a beneficial therapeutic option aimed at protecting myocardial function.

### Glucose metabolism disorder

The takotsubo heart is characterized by an overall decrease in myocardial glucose uptake and glycolytic rates in the hypocontractile areas during the acute phase of patients with TTS, and the metabolic abnormalities persist despite restoration of myocardial perfusion (Figure 2) (30, 31). Cardiac PET 18-flourodeoxyglucose, which reflect glucose utilization, has reported that impaired segments exhibit reduced glucose metabolism in the context of normal myocardial perfusion in TTS patients (32). Multiple investigators consider this flow-metabolism mismatch in patients with acute-stage TTS as the metabolic state of stunned myocardium, possibly owing to the fact that shifting levels of metabolites after myocardial stunning cause the decreased activity of key regulatory enzymes of the glycolysis pathway (32, 33). Another possibility is that high levels of circulating catecholamines mediated myocardial insulin resistance (IR) may disturb glucose utilization, thus decreasing glucose uptake (34). Observations that impaired glucose metabolism is related to the reduction of norepinephrine uptake in sympathetic overstimulation of takotsubo cardiomyopathy support this hypothesis (35). Indeed, glucose-insulin-potassium, regarded as a value metabolic intervention to treat TTS, has been reported to facilitate recovery from stunned myocardium by augmenting the amount of available glycolytic substrate (36). Likewise, cyclic adenosine monophosphate-mediated calcium overload and reduced sensitivity to calcium in the hypokinetic regions probably inhibit intracellular translocation of glucose transporter-4 (GLUT4), further contributing to reduced glucose uptake (37). However, animal models of TTS reveal significant increase in glucose uptake as well as metabolites but with reduced availability of final glycolysis metabolites and Krebs intermediates, which are less consistent in contrast to the above-mentioned clinical studies (6). By assessing substrate uptake and metabolism at various time points in the takotsubo rat heart, cardiac glucose uptake tends to increase initially followed by accumulation of glycolysis sugar phosphates, and remain modestly increased in the early compensated stage of TTS but decrease ultimately (21). The impaired glucose metabolism that possibly parallels cardiac dysfunction might be attributable in part to the proposed compensatory mechanism of a cross-regulation of glycolysis and beta-oxidation in an attempt by enhancing the more energy-efficient glycolysis and with the inhibition of β-oxidation via upregulation of gene/protein expression involved in cytoplasmic/mitochondrial glycolysis (6, 38). Therefore, although the early myocardial glucose uptake and its regulatory pathways are enhanced in TTS, the net result of myocardial metabolism is actual metabolite exhaustion and energetic deficit. Interestingly, takotsubo rats and the human condition during the acute phase are found to have increased glucose uptake driven by both cardiac myocytes and non-myocardial cells, such as the activated macrophages (39). In addition, Godsman et al. show upregulated GLUT1, which is primarily involved in cardiac stress responses and predominant isoform present in infiltrating macrophages, rather than the GLUT4 membrane transporter (6, 40). It is likely that the early overall upregulation of glycolysis pathway in the presence of decreased metabolites can also be interpreted by metabolically active macrophage infiltration whilst the absolute myocardial metabolism through glucose pathways actually reduces. The exact mechanism would require further differentiation between myocardial glucose uptake versus inflammatory macrophages during the different stages of acute TTS.

### Ketone bodies oxidation in takotsubo syndrome

Ketone bodies are primarily produced in the liver from acetyl-CoA and are metabolized in extra-hepatic tissue like myocardium (Figure 2) (41). When myocardial glucose utilization and FA metabolism are impaired, the main ketone body β-hydroxybutyrate (βOHB) is transported through the bloodstream into the heart and then oxidized into acetoacetate, which is further processed into acetyl-CoA utilized in the tricarboxylic acid (TCA) cycle. Two independent studies report that Ketone bodies become the preferred metabolic substrate in the heart in animal models and patients with TTS, indicating a more relevant energy source might be involved in ketone body oxidation in this setting (41, 42). Because the ketone body acetoacetate is preferred over FA by the myocardium according to the hierarchy of cardiac energy usage during acute stress, it favors an adaptive mechanism via ketone body oxidation over FA oxidation to elevate cardiac metabolic efficiency (41, 43). However, increased ketone oxidation suffers from the doubt whether it is accompanied by efficiency improvement in takotsubo hearts. Although the ketone oxidation-associated ketone body uptake and circulating levels of βOHB have been confirmed increased, ketone bodies may not significantly contribute to an increase in myocardial ATP production (44). Consequently, further research is necessary to understand whether the reliance on ketone body oxidation could be regarded as an adaptive or maladaptive response to improve takotsubo cardiac energy and has long-term benefits.

## Changes in cardiac effector subcellular structures in takotsubo syndrome

### Changes in cardiac mitochondrial function in takotsubo syndrome

#### Impaired oxidative metabolism

Takotsubo syndrome is accompanied by damages in mitochondrial oxidation, mainly including impaired ATP production, increased emission of ROS (45). As mentioned, the majority of ATP produced in a normal heart is due to the presence of mitochondrial oxidative capacity. The takotsubo heart is in a reduced mitochondrial oxidative state, which inhibits the coupling between the electron transport chain (ETC) system and phosphorylation reactions, leading in turn to a lessened ATP content than a healthy heart (46). Perturbations in ATP-generating processes may therefore trigger acute stress and the myocardial stunning, a paramount feature of TTS, and even develop a persistent, long-term HF phenotype (4). However, ATP deficiency is not the only relevant consequence of Impaired mitochondrial oxidation during TTS. Beyond the energetic aspect, alterations in cardiac oxidative stress may also implicate the development of TTS. ROS, such as superoxide and hydrogen peroxide, are byproducts of a coupled process between the TCA cycle and the ETC system and are indicators of mitochondrial stress and dysfunction (47). Emerging evidence suggests that increases in ROS are causally linked to the progression of TTS and cardiovascular outcomes of these patients (48). This notion is supported by expression profiling of cardiac genes in TTS patients showing the increase of Nrf2-driven ROS in causing myocardial stunning (49). Furthermore, Nef et al. demonstrated that endomyocardial biopsies from patients in the acute phase of TTS as compared to biopsy samples taken from controls or subjects after recovery had more myocardial superoxide production (50). A mechanistic link between oxidative stress and myocardial stunning has been revealed by *ex vivo* animal models in which ROS production and downstream inflammatory cells and inflammatory factors are significantly increased, subsequently inducing lipid peroxidation, damaging mitochondrial DNA, depleting antioxidants and further reducing ATP supply (51). Mechanistic studies also suggest that mitochondrial ROS emission is mainly associated with the activity of nicotinamide adenine dinucleotide phosphate (NADPH) oxidase (NOX) by overexpressing its subunits NOX2, NOX4, p47^phox^ and gp91^phox^ through mitogen-activated protein kinase (MAPK) signaling pathways, which contribute to the processes of cardiomyocyte apoptosis and necrosis (21, 51). Of note, inhibiting these effects of ROS by exogenous antioxidants, such as tempol and icariin, have been proven to be effective ways to protect against oxidative stress-induced cardiac dysfunction and prevent the progression of TTS in the acute phase compared with that of vehicle-treated animals (52, 53). In addition, Nrf2, as a transcription factor, has also been shown to active an upregulation of the antioxidant defense in the presence of ROS during the acute phase of TTS (49). This Nrf2-driven ROS detoxification pathway may suggest another possible antioxidative pathway to mitigate ROS-related cardiomyocyte damage.

#### Calcium mishandling

Mitochondrial redox state is directly linked to Ca^2+^ flux. To match energy supply with demand, the concentration of cytosolic Ca^2+^ elevates during increased workload (24). Although Ca^2+^ signaling is crucial for myocardial contractility, Ca^2+^ overload can trigger cell apoptosis or necrosis. This calcium mishandling has been substantiated by occasional contraction band necrosis of endomyocardial biopsies in clinical settings of TTS, which is largely associated with direct effects of catecholamines on cardiomyocytes viability through cyclic adenosine monophosphate (cAMP) mediated Ca^2+^ overload (26, 54). In TTS patients, the increased Ca^2+^ level observed in isolated inducible pluripotent stem cells is also in agreement with this finding. Furthermore, several investigations show alterations in genes expression of calcium metabolism-related proteins in TTS during the acute phase, the most important of which are sarcoplasmic reticulum (SR) Ca^2+^-adenosine-triphosphatase 2a (SERCA2a), sarcolipin, and phospholamban (55). In the takotsubo heart, SERCA2a gene expression is downregulated, whereas that of sarcolipin upregulated, accompanied by phospholamban dephosphorylation (54). Given that the capabilities of SR calcium reuptake and calcium stores are mainly determined by the phospholamban/SERCA2a ratio, the increased ratio decreases these properties and results in both systolic and diastolic dysfunction due to reduced Ca^2+^-affinity. At the mechanistic level, changes in gene expression are modulated by intense G-protein stimulated β_1_-adrenoceptor signaling through the cAMP responsive element binding protein-1 and nuclear factor of activated T-cells signaling pathways (55). By these alterations in cytosolic Ca^2+^ handling, depressed key dehydrogenases of the Krebs cycle and oxidation of NAD(P)H and flavin adenine dinucleotide (FAD) in experimental models of TTS are at the same timepoint as defective mitochondrial Ca^2+^ uptake, consequently driving the energetic mismatch in takotsubo cardiomyocytes. In agreement with these findings, studies in the model of lacking the mitochondrial Ca^2+^ uniporter also observe a compromise and delay in inotropic response to catecholamine stimulation (56). As a result, aberrant Ca^2+^ handling-mediated the energetic deficit might not be sufficient to sustain cardiac function in the setting of acute takotsubo cardiac dysfunction. Furthermore, the redox state of NADH and NADPH toward oxidation under this mismatch between energetic supply and demand might underlie the aggravation of mitochondrial ROS emission in TTS (6, 45). Meanwhile, mitochondrial-generated ROS could affect the SR Ca^2+^ release channel, which may form a positive feedback loop of mitochondrial dysfunction between calcium mishandling and oxidative stress after TTS and synergistically contribute to the progression of cardiac dysfunction (55).

Taken together, mitochondrial dysfunction, with the subsequent impairment in oxidative metabolism and Ca^2+^ mishandling, is a prominent determinant of impaired cardiac performance at the acute stage of TTS, and this finally may determine cardiomyocyte viability (Figure 2).

#### Endoplasmic reticulum stress in takotsubo syndrome

The endoplasmic reticulum (ER) has an important role in responding to inflammatory, metabolic disturbance, neurohumoral disorders and diverse stimuli that aggravate cardiomyopathy. An increment of the workload in the ER, known as ER stress, is accompanied by the accumulation of unfolded proteins in the lumen, known as the unfolded protein response (UPR) (57). TTS is closely associated with large amounts of catecholamine release, in which excessive ROS production and oxidative stress promotes cardiac injury, in part through ER stress and ER-associated apoptosis (58). It is well known that mitochondria as the main source of ATP is a crucial organelle governing oxidative stress and calcium turnover. Meanwhile, ER also produces small amounts of ROS, regulates cellular Ca^2+^ uptake, storage and signaling and maintains oxidizing redox potential in comparison with the cytosol, which are important for excitation-contraction coupling and apoptosis (57, 59). Changes in two homologous proteins sarcolipin and phospholamban bound to ER, which are crucial for intracellular calcium turnover and cardiac contractility, have been demonstrated in cardiac biopsies of TTS patients, causing the accumulation of cytoplasmic Ca^2+^ (60, 61). This Ca^2+^ overload leads to a further dysfunction in mitochondrial and cell death. *In vitro* studies combined with an analysis of myocardial biopsies from patients with TTS show that ER stress induces the expression of proteins such as glucose-regulated protein 78/immunoglobulin heavy chain binding protein, IRE1 and ATF6, which are involved in ER-mediated apoptosis through UPR ways, leading to foci of bound myocardial necrosis and the development of TTS (62). These findings suggest that targeting ER stress could offer a potential therapeutic strategy in the treatment and management of TTS.

## Changes in systemic metabolism in takotsubo syndrome

Although initially recognized as a myocardial metabolic inflexibility, TTS-associated metabolic dysfunction has been detected in the metabolite alterations from the serum or plasma of individuals with TTS and is now conceived as a systemic issue with complex interplay between the heart and peripheral tissues (63). Therefore, in addition to the changes in cardiac metabolism described above, the following sections outline widespread changes in systemic metabolism triggered by TTS, further leading to persistent long-term metabolic, functional and structural impairment in patients with TTS (Figure 1).

### Neurohumoral stress-response

Contemporary evidence strongly suggests that an involvement of the autonomic nervous system, the limbic network, and the renin-angiotensin-aldosterone system (RAAS) support an important role for the neurohumoral system in the pathophysiology of TTS, revealing a deep metabolic connection between the heart and brain.

The sympathetic overdrive remains the cornerstone for TTS pathogenesis with special emphasis on excessive local myocardial catecholamine levels and consequent activation of adrenoceptors in the heart. As evidence supporting increased concentrations of norepinephrine in the coronary sinus in patients with TTS becomes available, it suggests an increase in the local release of myocardial catecholamines and both the cardiac and systemic sympathetic systems that mediate the stress response (14, 63). Initial plasma catecholamine levels among patients with TTS are also several times those in age- and sex-matched patients with Killip class III myocardial infarction and remained substantially elevated even a week after the onset of symptoms. Additionally, increases in local norepinephrine levels released from sympathetic nerve terminals and circulating epinephrine and norepinephrine released from adrenal medullary chromaffin cells have been demonstrated in the acute phase of TTS which might be mediated by neuronally transmitted norepinephrine, further suggesting the activation of the adrenomedullary hormonal system (64). Moreover, TTS patients have evidence of elevated plasma levels of neuropeptide Y, which is stored in postganglionic sympathetic nerves and adrenal chromaffin cells and released in response to stress, along with significantly elevated plasma levels of brain natreuretic peptide and serotonin (63). The enhanced activity of this neuroendocrine stress-response axis is crucial to maintain high levels of stress arousal and related-hormonal output for prolonged periods. Given the central role of sympathetic neurohormonal metabolism in TTS and its well-described associations with clinical outcomes, interest in sympathetic metabolism disorders as a potential treatment target and prognostic value in TTS has increased in recent years.

Recently, the limbic network has been discovered that can mediate the vegetative and endocrine functions of the body during stress conditions of TTS (65). By administering the glucose analog 18F-FDG in the acute phase of TTS, higher neural activity appears in the hippocampus and the other components of the limbic system, which manifests as an increased neuronal glucose metabolism (66). In contrast, decreased metabolism is observed in the pre-frontal cortex during periods of acute mental or physical stress (66, 67). These metabolic alterations were further substantiated by a follow-up study, in which changes in the limbic system appear to persist even after full recovery of cardiac function in TTS.

Finally, the metabolic activity of RAAS is well-represented in both heart and brain, where it becomes activated resulting in elevated plasma neurohormone concentrations in response to acute heart injury and further decompensation. There is evidence that long-term maladaptive activation of RAAS persists in TTS despite “normalization” of LV ejection fraction (LVEF) (68). It further provides a basis for the clinical management of reduction in angiotensin II levels to improve long-term outcomes in TTS patients.

In addition to supporting the clinical relationship between neuroendocrine stimulation and myocardial stunning, the mechanism underlying this association is complex. Beyond the catecholamine-mediated direct myocyte injury, the effects of catecholamine storm systemically, and particularly upon peripheral vasculature, should also be focused on. Extensive research has identified both the central and autonomic nervous systems mediate the stress response in the pathophysiology of TTS (64, 69). As a central principal site for the synthesis of norepinephrine, the activation of locus coeruleus trigger noradrenergic responses after the initial sympathetic overdrive, which in turn stimulates the hypothalamic-pituitary-adrenal axis (3, 14). The result is a surge rapidly in circulating catecholamines, with an afterload dependent mechanism resulting in epicardial and coronary arterial spasm, microvascular dysfunction via the β-adrenoceptor–mediated cAMP-dependent protein kinase pathway and contributing to the development of TTS (3). Notably, from multiple observations, changes of endothelial dysfunction might constitute a strong association between this catecholamine surge and myocardial ischemia, especially in estrogen deficiency-postmenopausal women in TTS. Subsequently validated in an experimental study, Estrogen supplementation largely prevents the stress-induced apical stunning via endothelium-dependent and other independent mechanisms and hypothalamo-sympatho-adrenal outflow from the brain to the target organs (64). Further investigation is needed to clarify the relationship of neuroendocrine stress-response in TTS and whether these responses are pre-existing and contribute to the onset of TTS or are acquired as a consequence of the initial sympathetic storm and TTS episode.

### Insulin resistance

Traditionally recognized as a defining metabolic feature of type 2 diabetes and metabolic syndrome, IR is also a central metabolic manifestation in TTS. Present in most of non-diabetic TTS patients, IR coincides with glucose and lipid metabolism disturbance, defective mitochondrial oxidative metabolism and elevated circulating inflammatory marker (36). Additionally, insulin specifically has been identified to have protective effects on left ventricular contractility and hemodynamics to improve heart function in animal models of catecholamine-induced cardiomyopathy and patients with TTS (36, 70). Since the prevalence of IR in TTS and the well-described association between insulin and pathophysiology, treatment and prognosis of TTS, it appears to be plausible for insulin blood levels as a diagnostic biomarker, which is inversely paralleled with the severity of TTS.

The relationship between IR and TTS initially results from high levels of circulating catecholamines. Researchers reveal significant decreases in pancreatic insulin release and peripheral insulin sensitivity as a consequence of catecholamines overdrive, finally contributing to impaired lipid, glucose and related metabolites utilization of myocardium (27, 70). Subsequently validated in human TTS cohorts, this IR-associated myocardial metabolism variation has also been reported based on metabolomics research results (36). More importantly, these glycolipid changes further result in the development of IR through inducing post-translational modifications of several key factors of the insulin signaling cascade (31). In addition to heightened inflammatory signaling, the mechanistic link between the two is now widely accepted that metabolic abnormalities can phosphorylate insulin receptor substrate (IRS)-1 on serine residues by activating several “stress” kinases, which are major contributors to whole body IR (71). Therefore, improving IR may be a beneficial mechanism aimed at protecting the ischemic myocardium during apical ballooning from the detrimental effects of substrate disorder. In fact, several lines of investigation have been shown that insulin could stimulate glucose uptake in stunned myocardium to reverse the condition of myocardial metabolism from FAs to preferential carbohydrate oxidation for improving cardiac action and metabolic complications (31, 36).

### Catabolic: Anabolic imbalance

Another common pathophysiologic process of TTS, which significantly impacts the systemic metabolic phenotype, is a shift of metabolic balance toward hypercatabolism, with impairment of the anabolic phase (72). Evidence suggests that excess catecholamines lead to accumulation of metanephrine and normetanephrine of extraneuronal catecholamine metabolites in the circulation, impairments in FA oxidation and increasement of plasma levels of free FA (63). Additionally, norepinephrine overstimulation influences the net cellular synthesis of phosphoenolpyruvate carboxykinase (PEPCK) and glucose-6-phosphatase (G6Pase), two essential enzymes for the anabolic metabolism of glucose, via the β_2_-adrenergic receptor signaling pathway, thus blunting gluconeogenesis (63, 72). Aside from the concentration of inhibitory or stimulatory metabolites and the degree of expression and allosteric modification of key regulatory enzymes, changes in metabolic proteins especially transporters, also rapidly control the flux and balance of metabolism pathways in the setting of acute stresses. In 2013, Shao et al. identify the depressed gene expression of ApoB lipoprotein and subsequent decreased lipid export that correlate significantly with cardiac lipotoxicity and downregulation of FA uptake and its oxidative metabolism (22). Since by-products of lipid accumulation or other relevant metabolic intermediates could be released into the plasma compartment, a peripheral blood metabolite signature of hypercatabolism may be detected with metabolomics investigations.

Notably, with elongation of the catabolic phase over the anabolic phase, this metabolic shift causes a depletion of energy storage in cardiomyocytes and a reduced turnover of cell constituents in patients with TTS. Specifically, Kofron et al. find that cell interactions are affected, as a decrease in coupling between cardiomyocytes while an increase in coupling between cardiomyocytes and fibroblasts (73). These observations may ultimately result in alterations in the shape and velocity of action potential predisposing to arrhythmias, fibroblast activation and deposition of extracellular matrix promoting cardiac remodeling, further promoting the development of takotsubo cardiomyopathy.

## Long-term metabolic changes after takotsubo syndrome

Despite TTS used to be considered as a merely acute benign condition, recent evidence raise concerns about the long-term clinical and functional consequences of TTS even after the apparent rapid restoration of LVEF and cardiac wall motion abnormalities. Studies in a prospectively recruited cohort of patients have reported persistence of myocardial edema associated with impaired cardiac energetic metabolism together with a process of global microscopic fibrosis at 4-month follow-up, resulting in the possibility of developing HF with preserved EF (74, 75). Given that sufficient energy supply is essential for cardiac functional recovery, a longer convalescent period or incomplete restoration would be suggested to be responsible for adverse long-term prognosis at a comparable level of that of myocardial infarction. Dawson et al. (74) conduct an observational cross-sectional case-control research of patients with prior (>12 months) TTS and demonstrate that after TTS, the impairment of cardiac energetic status and reduction of maximal oxygen consumption on exercise, which is consistent with their metabolic testing, are ongoing and contribute to a persistent HF phenotype with a significant impact on quality of life (4). Of note, the majority (88%) of patients enrolled in the study have long-term symptoms compatible with HF at even 20 (range 13–39) months after the occurrence of TTS, as confirmed by a median score of 13 in the Minnesota Living with Heart Failure Questionnaire (4). Although a previous recognized establishment of clinical features identifying those at risk, the metabolic characteristics of these patients long term described in this study, including lower peak oxygen consumption and higher slope of the minute ventilation/carbon dioxide production relationship, are predictors of adverse cardiovascular outcome in HF of several etiologies and may also be features of post-TTS. The findings of abnormal metabolic performance in patients with prior TTS are perhaps surprising and puzzling, but these abnormalities could begin to explain the nature of this long-term state of worse outcomes with preserved LVEF, at least in part, and might point to a potential therapeutic target in those cases with a poor clinical consequence. There are currently two possible explanations: given the link between myocardial substrate metabolism and cardiac energetics, a switch in substrate preference away from carbohydrate oxidation toward FA oxidation in the takotsubo status, which might increase oxygen consumption further, could be associated with impaired energy mechanisms (33). Another possibility is that a secondary and persistent extracardiac neuroendocrine condition in TTS via a humoral or anatomical brain-heart axis even become a major contributor, or coexist with the above, causing adverse long-term prognosis (76). Thus, prospective studies on cardiac and extracardiac metabolic alterations in the acute phase and systematic followup, including the cause of death, of TTS patients are important because they may provide not only a better mechanistic understanding and a new target for therapeutic intervention but also valuable prognostic information to unmask intrinsic heart dysfunction beyond EF.

## Diagnostic workup

### General diagnostic algorithm

**FIGURE 3:**
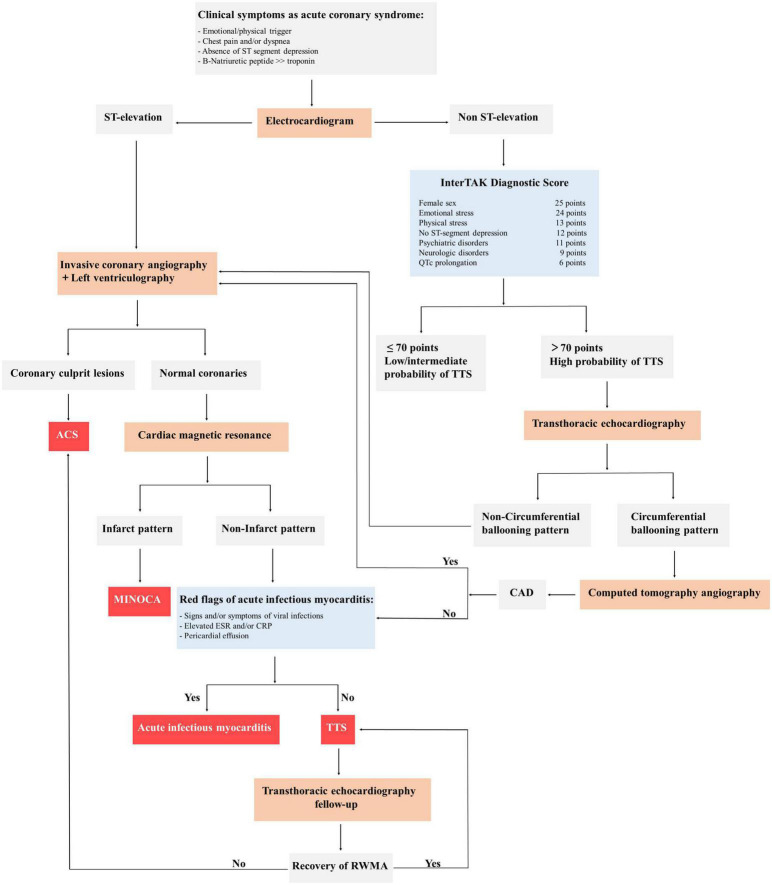
General diagnostic algorithm of takotsubo syndrome (TTS). ACS, acute coronary syndrome; CAD, coronary artery disease; MINOCA, myocardial infarction with non-obstructed coronary arteries; InterTAK, International Takotsubo Registry; QTc, QT-time corrected for heart rate; ESR, erythrocyte sedimentation rate; CRP, c-reactive protein; RWMA, regional wall motion abnormality; TTS, takotsubo syndrome.

The diagnosis of TTS is generally based on clinical features in combination with the absence of coronary stenosis and the regional wall-motion abnormalities beyond the territory perfused by a single epicardial coronary artery (34). In patients with acute chest pain and ST-segment elevation urgent diagnostic coronary angiography must be considered to exclude acute myocardial infarction. Patients presenting with stable condition and non-ST-segment elevation could perform CT coronary angiography as an alternative imaging investigation or the International Takotsubo Registry (InterTAK) Diagnostic Score. According to this tool, an InterTAK Score ≤ 70 indicates a low probability for the presence of TTS, while an InterTAK Score ≥ 70 suggests a probability of stress cardiomyopathy of 90% (77). Coronary angiography with left ventriculography should be considered in patients who present with a low probability, while transthoracic echocardiography could be useful in patients with a high score. Of note, in case of positive signals of acute infectious myocarditis, cardiac magnetic resonance imaging should be recommended to exclude infectious myocarditis and confirm the diagnosis of TTS (Figure 3).

### Potential metabolic diagnostic approach for takotsubo syndrome

Several studies have focused on the potential utility of metabolic imaging, although it has been performed mainly for research purpose to provide additional pathophysiologic information about TTS and has not been determined in the clinical setting. Kurisu et al. (25) note that impairment of myocardial fatty acid metabolism appears to be more severe than myocardial perfusion by using tallium-201 (201Tl) and iodine123-β-Methyl-piodophenyl penta-decanoic acid (123I-BMIPP) dual-isotope myocardial SPECT in 14 postmenopausal women with TTS. More importantly, this metabolic impairment persists during follow-up, even after resolution of myocardial perfusion abnormality and the systolic dysfunction. Similarly, following TTS patients for a longer time, Sato et al. (78) demonstrate a marked perfusion-fatty acid metabolism mismatch in the apical myocardium improved with 6 months. Furthermore, Owa et al. (79) perform serial comparative metabolic and sympathetic nervous imaging in four patients with TTS after the acute event, even though disturbance of cardiac sympathetic innervation persists for 1 year after the improvement of the defective metabolism and perfusion. Recently, in TTS patients who suffered an acute episode > 1 year previously, it has also been shown that reduced cardiac energetic status is in keeping with their exercise and metabolic performance testing (4). Therefore, metabolism-related diagnostic approaches may provide incremental value for the differential and correct diagnosis of TTS, and facilitate therapeutic decision-making in suspected individuals.

## Treatment options

### General considerations

To date, there are no randomized clinical trial data or specific guidelines to define the optimal management of TTS patients. Therefore, management algorithms are based on clinical experience and expert consensus. In mild cases with no hemodynamic instability and pulmonary congestion, a short course of diuretic agents and vasodilators to relieve congestion may be sufficient. If hypertension exists, arterial vasodilators and β-Adrenergic blockers are useful. In severe cases complicated by cardiogenic shock, treatment depends on the presence of haemodynamically significant LV outlet tract obstruction (LVOTO). In patients with no LVOTO, inotropic agents, and if not sufficient, early temporary LV assist devices (LVADs) should be considered. In the presence of LVOTO, extracorporeal membrane oxygenation or LVADs as a bridge-to-recovery for refractory cases of TTS with LVOTO or biventricular failure is recommended. If early mechanical support is not available, low dose of levosimendan infusion as a catecholamine-sparing positive inotrope may be with caution. New trial evidence is required to assess the safety and efficacy of levosimendan in these complex patients.

### Current metabolic therapy for takotsubo syndrome

Given that abnormally high sympathetic responses are accepted to have a central role in the pathophysiology of TTS, various types of β-Blockers are often being used acutely and after discharge of patients with TTS. During the acute phase, several clinical cases have demonstrated that β-Blockers, like metoprolol, propranolol and esmolol, are particularly useful in cases with TTS complicated by LVOTO (1, 80). Furthermore, propranolol and phentolamine can prevent necrotic myocardial lesions based on case reports of sympathetic overactivation, whereas necrotic lesions occur in all six patients who received a placebo (81). Of note, β-Blockers have no favorable role on in-hospital mortality in TTS patients with or without LVOTO (82). Indeed, the abrupt withdrawal of metoprolol may even trigger TTS in individual cases (80). For the long-term therapy, β-Blockers, as the most commonly prescribed medication at hospital discharge, have been found to have a protective effect against TTS recurrence (80). Similarly, Labetalol is also deemed valuable in the prevention of recurrent TTS induced by electroconvulsive therapy for depression. However, some observational studies do suggest no beneficial association between long-term β-Blocker use and survival at 1 year in patients with TTS, while angiotensin-converting enzyme inhibitors or angiotensinreceptor blockers are beneficial for survival after propensity matching (83). The authors also report that one-third of patients experience a TTS recurrence during the pharmacotherapy of β-Blockers. As evidences supporting the cardiotoxic effect of high norepinephrine levels through binding to α 1-Adrenergic receptors and the feasibility research of treatment with a combined α- and β-Blocker in the animal model become available (84), carvedilol and labetalol, which both possess the blocker characteristics of non-selective β-Adrenergic receptor and α1-adrenergic receptor, could be considered more for the acute and chronic management of TTS patients. Future randomized clinical trials should certainly evaluate the efficacy, dose, and type of β-Blockers for the treatment of TTS and the prevention of TTS recurrence at follow-up.

Additionally, in contrast to previous perceptions, TTS has long-lasting structural and metabolic alterations associated with a persistent, long-term HF phenotype. Considering the improvements in cardiac function and myocardial energetics of long-term therapy with β-Adrenergic receptor antagonists in HF patients (85), it could be worth identifying the long-lasting regulation mechanisms and metabolic action of β-Blockade or other potential metabolic therapy to better improve the final outcome of patients with TTS. The remaining standard therapies, like mechanical support for acute cardiogenic shock and anticoagulation for patients with severe LV dysfunction, are also thought to indirectly and partly improve cardiac metabolism by diminishing the mechanical workload, thereby sparing the heart energy.

### Potential metabolic therapy for takotsubo syndrome

Growing metabolism-related studies serve as important proofs of principle for the application of metabolic modulators to TTS, although there are currently poor understandings of the metabolic regulatory mechanisms over the course of TTS and no definitive and large-scale controlled clinical trials proving the effectiveness of these metabolic or mitochondrial therapeutic intervention for patients with TTS. Given that guidelines available for the management of TTS are absent, therapeutic strategies with the recent evidence from small clinical and animal researches, in particular for targeting substrate utilization and/or oxidative stress, might be promising tools to improve the outcome of patients with TTS beyond that achieved with traditional sympathetic inhibition and symptomatic therapies.

### Targeting substrate metabolism in takotsubo syndrome

Despite our expanding knowledge of metabolic alterations associated with takotsubo cardiomyopathy, especially the simultaneous dysregulation of glycolytic and beta-oxidation pathways, therapeutic interventions targeting substrate metabolism to manage TTS are lacking in the clinical arena. In terms of a mechanism, myocardial substrate metabolism in the takotsubo heart has been suggested at least a degree of shifting away from FA utilization to preferential carbohydrate oxidation, and thus, further stimulation of glucose myocardial uptake is hypothesized to be beneficial to cardiac bioenergetics, hemodynamics, and restoration of cardiac function. In addition, this approach could also balance overall glucose metabolism between glycolysis and glucose oxidation (27). Indeed, the optimal effects of insulin treatment have been identified in stunned myocardium in light of myocardial IR associated with excessive catecholamine stimulation in TTS with or without diabetes (36). Moreover, insulin therapy combined with infusion of dextrose and potassium solutions and use of β-Adrenoceptor blockers, which could enhance the effectiveness of insulin, improve cardiac performance, exercise capacity, and symptoms, so the complete therapeutic managements should be applied as early as possible in the progress of TTS (36). Another metabolic strategy, estrogen supplementation, increases cardiac glucose uptake and insulin sensitivity, upregulates the cardiac levels of cardioprotective substances and modestly improves LVEF in the human heart (37). However, considering that estrogen replacement is a required condition, but not completely adequate for the treatment of patients with TTS, the detailed mechanism and the viability of this therapeutic approach, especially based on restoring glucose metabolism, are still being questioned. Indeed, the real beneficial effects of estrogen supplementation may be independent of a mild augmentation in cardiac glucose utilization and may instead contribute to alleviating the side effects of increased sympathetic activity on target organs in synergy with insulin therapy and β-Blockers. Therefore, a better understanding of the metabolic link between estrogen and glucose utilization, or whether the time gap exists between this therapeutic action and its full efficacy are needed to disclose.

An alternative therapeutic option is the use of PARP-1 inhibitors for takotsubo cardiomyopathy, which could favorably modulate the deranged metabolism of myocardial FA oxidative pathways by increasing their utilization (6). Furthermore, treatment with PARP-1 inhibitors ameliorate nitrosative stress to further improve energetic impairment in the setting of TTS, but clinical trials investigating their effects remain unsettled (6). In addition, increasing FA uptake with overexpression of ApoB, a myocardial lipid-exporting protein, is found to prevent lipid accumulation and takotsubo-like cardiac dysfunction in mouse models, which is consistent with the evidence that suggests downregulated gene expression of ApoB in the akinetic myocardium is displayed in TTS patients during the acute phase (22). Although the promising results just demonstrated in animal models, these findings suggest that lipid transport system could be a potential therapeutic target and clinical studies should test this in the condition of TTS for which no optimal treatment currently exists.

### Targeting mitochondrial reactive oxygen species

Modulation of ROS specifically in mitochondria has been a potential target in TTS, given the crucial role of ROS involved in impaired cardiac energetics and metabolic and structural remodeling in progression of takotsubo heart. Hydrogen sulfide (H_2_S) is a physiological component mainly produced via cystathionine γ-Lyase (CSE) in the heart, exerting cardioprotective effects on oxidative stress reaction by reducing NADPH oxidase mediated ROS production, thereby functioning as a ROS scavenger (86). In TTS, plasma and myocardial levels of H_2_S are significantly decreased, are related to the severity of the disease, while exogenous treatment with sodium hydrosulfide not only recovers the H_2_S levels and CSE activity, but also ameliorates cardiac dysfunction and reduces mortality (86, 87). However, because this therapeutic target is underpowered, which has not yet been tested in TTS patients, caution is warranted when discussing H_2_S administration in the current guidelines for the treatment of patients with TTS. As other antioxidants, especially α-Lipoic acid, have been proven to be an effective way to ameliorate myocardial dysfunction and remodeling by directly scavenging free radicals and improving antioxidant defense system in TTS patients, future studies and clinical practice should paid enough attention to further translate these mitochondria- targeted antioxidants into clinical conditions (88).

Nrf2 is a transcription factor that actives transcriptional control genes, several antioxidant response elements such as ascorbic acid, SOD, catalase and GPX1, in the presence of ROS (47, 49). Without being a direct antioxidant, Nrf2 nevertheless decreases mitochondrial oxygen stress most likely by driving ROS detoxification pathway, which might be the most powerful endogenous antioxidative signaling pathway to prevent ROS formation in the acute phase of TTS. Nrf2 ameliorates TTS development in various animal models of takotsubo heart, may be safe in TTS patients, and should expand clinic data available from patients with TTS. Given that ROS also have a functional modulation role in modulating Ca^2+^ signaling pathway, targeting the compromised Ca^2+^ release might be an alternative approach. As recently discovered, restoring the abnormalities in cytosolic and mitochondrial Ca^2+^ handling by Ca^2+^-sensitizer levosimendan as an alternative inotrope to catecholamine agent might be promising approaches (89). Finally, increasing SR Ca^2+^ release and reuptake would prevent cardiac hypercontraction, maladaptive cardiac remodeling and Ca^2+^-linked arrhythmias in a rat model of TTS (22). However, clinical application and translation of these ways have not yet been attempted.

## Faced challenges and future directions

Despite the substantial increment in recognition of TTS by the medical community, there remain several knowledge gaps in this complex syndrome. Perhaps the most urgent is to focus on the pathophysiological mechanisms and characteristics involved, as well as the long-term clinical progress of Takotsubo patients, to firmly establish disease-specific management guidelines that allow clinicians to diagnose this syndrome with certainty and to treat in a timely manner. Exploring a sensitive and specific diagnostic test containing the assessment of risk factors, clinical features, imaging manifestations, biochemical markers, and even metabolic changes might be beneficial to increase the accuracy of the diagnosis and identify the risk stratification and prognostication. In terms of therapeutic options, not only does the management of the acute phase of TTS needs to be further defined, especially guidance on the effective therapy of severe complications, but the long-term care of EF-recovered TTS, especially in those patients who continue to have symptoms and are prone to recurrent adverse cardiac and cerebrovascular events is also crucial. Given the presence of cardiac energetic impairment and systemic metabolic disorders, a potential role for targeting these metabolic impairments may be beneficial in the treatment of TTS. Further randomized and adequately powered studies of specific substrate use and improvement of metabolism are an attractive investigative pathway to translate presumed theoretical modalities into clinical efficacy.

## Conclusion

In conclusion, metabolic remodeling in TTS is involved in profound changes of a complex and tightly regulated network, which is deranged at several levels: sympathetic metabolism, substrate metabolism, effector subcellular dysfunction, redox regulation, and systemic metabolism, collectively contributing to the progression of the disease and the development of a persistent and long-term HF phenotype. Therefore, in the takotsubo heart, modulating this mechanistic core may improve critical metabolic processes, cardiac function, and systematic disorders with long-term benefits on quality of life in patients. Nevertheless, more progress needs to be made in the exact mechanisms linking metabolic derangements to other pathophysiology in TTS. Future mechanistic investigations are warranted to decipher this complex network and translate this knowledge into viable therapeutic approaches for TTS.

## Author contributions

TW researched the data for the manuscript and wrote the manuscript with TX. YY and DW discussed the content of the manuscript and reviewed before submission. BZ and XC helped with the literature search and literature collation. All authors contributed to the article and approved the submitted version.

## References

[1] Medina de Chazal H Del Buono MG Keyser-Marcus L Ma L Moeller FG Berrocal D Stress cardiomyopathy diagnosis and treatment: JACC state-of-the-art review. *J Am Coll Cardiol.* (2018) 72:1955–71. 10.1016/j.jacc.2018.07.072 30309474 PMC7058348

[2] Kastaun S Gerriets T Tschernatsch M Yeniguen M Juenemann M. Psychosocial and psychoneuroendocrinal aspects of Takotsubo syndrome. *Nat Rev Cardiol.* (2016) 13:688–94. 10.1038/nrcardio.2016.108 27411402

[3] Lyon AR Citro R Schneider B Morel O Ghadri JR Templin C Pathophysiology of Takotsubo syndrome: JACC state-of-the-art review. *J Am Coll Cardiol.* (2021) 77:902–21. 10.1016/j.jacc.2020.10.060 33602474

[4] Scally C Rudd A Mezincescu A Wilson H Srivanasan J Horgan G Persistent long-term structural, functional, and metabolic changes after stress-induced (Takotsubo) cardiomyopathy. *Circulation.* (2018) 137:1039–48. 10.1161/CIRCULATIONAHA.117.031841 29128863 PMC5841855

[5] Yoshida T Hibino T Kako N Murai S Oguri M Kato K A pathophysiologic study of tako-tsubo cardiomyopathy with F-18 fluorodeoxyglucose positron emission tomography. *Eur Heart J.* (2007) 28:2598–604. 10.1093/eurheartj/ehm401 17921529

[6] Godsman N Kohlhaas M Nickel A Cheyne L Mingarelli M Schweiger L Metabolic alterations in a rat model of Takotsubo syndrome. *Cardiovasc Res.* (2022) 118:1932–46. 10.1093/cvr/cvab081 33711093 PMC9239582

[7] De Jong KA Lopaschuk GD. Complex energy metabolic changes in heart failure with preserved ejection fraction and heart failure with reduced ejection fraction. *Can J Cardiol.* (2017) 33:860–71. 10.1016/j.cjca.2017.03.009 28579160

[8] Schiattarella GG Rodolico D Hill JA. Metabolic inflammation in heart failure with preserved ejection fraction. *Cardiovasc Res.* (2021) 117:423–34. 10.1093/cvr/cvaa217 32666082 PMC8599724

[9] Kimball TH Vondriska TM. Metabolism, epigenetics, and causal inference in heart failure. *Trends Endocrinol Metab.* (2020) 31:181–91. 10.1016/j.tem.2019.11.009 31866216 PMC7035178

[10] Lopaschuk GD. Metabolic modulators in heart disease: past, present, and future. *Can J Cardiol.* (2017) 33:838–49. 10.1016/j.cjca.2016.12.013 28279520

[11] Stanley WC Recchia FA Lopaschuk GD. Myocardial substrate metabolism in the normal and failing heart. *Physiol Rev.* (2005) 85:1093–129. 10.1152/physrev.00006.2004 15987803

[12] Mather KJ Hutchins GD Perry K Territo W Chisholm R Acton A Assessment of myocardial metabolic flexibility and work efficiency in human type 2 diabetes using 16-[18F]fluoro-4-thiapalmitate, a novel PET fatty acid tracer. *Am J Physiol Endocrinol Metab.* (2016) 310:E452–60. 10.1152/ajpendo.00437.2015 26732686 PMC4796267

[13] Taegtmeyer H Golfman L Sharma S Razeghi P van Arsdall M. Linking gene expression to function: metabolic flexibility in the normal and diseased heart. *Ann N Y Acad Sci.* (2004) 1015:202–13. 10.1196/annals.1302.017 15201161

[14] Pelliccia F Kaski JC Crea F Camici PG. Pathophysiology of Takotsubo syndrome. *Circulation.* (2017) 135:2426–41. 10.1161/CIRCULATIONAHA.116.027121 28606950

[15] Basso C Thiene G. The pathophysiology of myocardial reperfusion: a pathologist’s perspective. *Heart.* (2006) 92:1559–62. 10.1136/hrt.2005.086959 16547203 PMC1861239

[16] Sardu C Sacra C Mauro C Siniscalchi M Marfella R Rizzo MR. 123I-MIBG scintigraphy in the subacute state of Takotsubo cardiomyopathy. *JACC Cardiovasc Imaging.* (2017) 10:93–4. 10.1016/j.jcmg.2016.07.016 28057224

[17] Nef HM Möllmann H Kostin S Troidl C Voss S Weber M Tako-Tsubo cardiomyopathy: intraindividual structural analysis in the acute phase and after functional recovery. *Eur Heart J.* (2007) 28:2456–64. 10.1093/eurheartj/ehl570 17395683

[18] Paur H Wright PT Sikkel MB Tranter MH Mansfield C O’Gara P High levels of circulating epinephrine trigger apical cardiodepression in a β2-adrenergic receptor/Gi-dependent manner: a new model of Takotsubo cardiomyopathy. *Circulation.* (2012) 126:697–706. 10.1161/CIRCULATIONAHA.112.111591 22732314 PMC4890655

[19] Akashi YJ Nef HM Lyon AR. Epidemiology and pathophysiology of Takotsubo syndrome. *Nat Rev Cardiol.* (2015) 12:387–97. 10.1038/nrcardio.2015.39 25855605

[20] Chen W Dilsizian V. Cardiac sympathetic disturbance in Takotsubo cardiomyopathy: primary etiology or a compensatory response to heart failure? *JACC Cardiovasc Imaging.* (2016) 9:991–3. 10.1016/j.jcmg.2016.01.026 27344419

[21] Borchert T Hübscher D Guessoum CI Lam TD Ghadri JR Schellinger IN Catecholamine-dependent beta-adrenergic signaling in a pluripotent stem cell model of Takotsubo cardiomyopathy. *J Am Coll Cardiol.* (2017) 70:975–91. 10.1016/j.jacc.2017.06.061 28818208

[22] Shao Y Redfors B Ståhlman M Täng MS Miljanovic A Möllmann H A mouse model reveals an important role for catecholamine-induced lipotoxicity in the pathogenesis of stress-induced cardiomyopathy. *Eur J Heart Fail.* (2013) 15:9–22. 10.1093/eurjhf/hfs161 23099354

[23] Lyon A. Stress in a dish: exploring the mechanisms of Takotsubo syndrome. *J Am Coll Cardiol.* (2017) 70:992–5. 10.1016/j.jacc.2017.07.716 28818209

[24] Bai P Cantó C Oudart H Brunyánszki A Cen Y Thomas C PARP-1 inhibition increases mitochondrial metabolism through SIRT1 activation. *Cell Metab.* (2011) 13:461–8. 10.1016/j.cmet.2011.03.004 21459330 PMC3086520

[25] Kurisu S Inoue I Kawagoe T Ishihara M Shimatani Y Nishioka K Myocardial perfusion and fatty acid metabolism in patients with Tako-Tsubo-like left ventricular dysfunction. *J Am Coll Cardiol.* (2003) 41:743–8. 10.1016/s0735-109702924-8 12628716

[26] Ghadri JR Wittstein IS Prasad A Sharkey S Dote K Akashi YJ International expert consensus document on Takotsubo syndrome (part I): clinical characteristics, diagnostic criteria, and pathophysiology. *Eur Heart J.* (2018) 39:2032–46. 10.1093/eurheartj/ehy076 29850871 PMC5991216

[27] Hantson P Beauloye C. Myocardial metabolism in toxin-induced heart failure and therapeutic implications. *Clin Toxicol.* (2012) 50:166–71. 10.3109/15563650.2012.658472 22335503

[28] Fineschi V Michalodimitrakis M D’Errico S Neri M Pomara C Riezzo I Insight into stress-induced cardiomyopathy and sudden cardiac death due to stress. A forensic cardio-pathologist point of view. *Forensic Sci Int.* (2010) 194:1–8. 10.1016/j.forsciint.2009.10.025 19939595

[29] Chiu HC Kovacs A Blanton RM Han X Courtois M Weinheimer CJ Transgenic expression of fatty acid transport protein 1 in the heart causes lipotoxic cardiomyopathy. *Circ Res.* (2005) 96:225–33. 10.1161/01.RES.0000154079.20681.B9 15618539

[30] Ibrahim T Nekolla SG Langwieser N Rischpler C Groha P Laugwitz KL Simultaneous positron emission tomography/magnetic resonance imaging identifies sustained regional abnormalities in cardiac metabolism and function in stress-induced transient midventricular ballooning syndrome: a variant of Takotsubo cardiomyopathy. *Circulation.* (2012) 126:e324–6. 10.1161/CIRCULATIONAHA.112.134346 23169256

[31] Kakinuma Y Okada S Nogami M Kumon Y. The human female heart incorporates glucose more efficiently than the male heart. *Int J Cardiol.* (2013) 168:2518–21. 10.1016/j.ijcard.2013.03.016 23545149

[32] Rendl G Rettenbacher L Keinrath P Altenberger J Schuler J Heigert M Different pattern of regional metabolic abnormalities in Takotsubo cardiomyopathy as evidenced by F-18 FDG PET-CT. *Wien Klin Wochenschr.* (2010) 122:184–5. 10.1007/s00508-010-1356-7 20361383

[33] Obunai K Misra D Van Tosh A Bergmann SR. Metabolic evidence of myocardial stunning in Takotsubo cardiomyopathy: a positron emission tomography study. *J Nucl Cardiol.* (2005) 12:742–4. 10.1016/j.nuclcard.2005.06.087 16344237

[34] Ghadri JR Wittstein IS Prasad A Sharkey S Dote K Akashi YJ International expert consensus document on Takotsubo syndrome (part II): diagnostic workup, outcome, and management. *Eur Heart J.* (2018) 39:2047–62. 10.1093/eurheartj/ehy077 29850820 PMC5991205

[35] Cimarelli S Sauer F Morel O Ohlmann P Constantinesco A Imperiale A. Transient left ventricular dysfunction syndrome: patho-physiological bases through nuclear medicine imaging. *Int J Cardiol.* (2010) 144:212–8. 10.1016/j.ijcard.2009.04.025 19443060

[36] Madias JE. Insulin and Takotsubo syndrome: plausible pathophysiologic, diagnostic, prognostic, and therapeutic roles. *Acta Diabetol.* (2021) 58:989–96. 10.1007/s00592-021-01709-7 33811293

[37] Vitale C Rosano GM Kaski JC. Role of coronary microvascular dysfunction in Takotsubo cardiomyopathy. *Circ J.* (2016) 80:299–305. 10.1253/circj.CJ-15-1364 26763468

[38] Krishnan J Suter M Windak R Krebs T Felley A Montessuit C Activation of a HIF1alpha-PPARgamma axis underlies the integration of glycolytic and lipid anabolic pathways in pathologic cardiac hypertrophy. *Cell Metab.* (2009) 9:512–24. 10.1016/j.cmet.2009.05.005 19490906

[39] Wilson HM Cheyne L Brown PAJ Kerr K Hannah A Srinivasan J Characterization of the myocardial inflammatory response in acute stress-induced (Takotsubo) cardiomyopathy. *JACC Basic Transl Sci.* (2018) 3:766–78. 10.1016/j.jacbts.2018.08.006 30623136 PMC6314973

[40] Fillmore N Mori J Lopaschuk GD. Mitochondrial fatty acid oxidation alterations in heart failure, ischaemic heart disease and diabetic cardiomyopathy. *Br J Pharmacol.* (2014) 171:2080–90. 10.1111/bph.12475 24147975 PMC3976623

[41] Nanda S Longo S Bhatt SP Pamula J Sharma SG Dale TH. Stress cardiomyopathy – A unique presentation of diabetic ketoacidosis. *Ann Clin Biochem.* (2009) 46(Pt 3):257–60. 10.1258/acb.2009.008237 19307252

[42] Lopaschuk GD Karwi QG Ho KL Pherwani S Ketema EB. Ketone metabolism in the failing heart. *Biochim Biophys Acta Mol Cell Biol Lipids.* (2020) 1865:158813. 10.1016/j.bbalip.2020.158813 32920139

[43] Zhang Y Taufalele PV Cochran JD Robillard-Frayne I Marx JM Soto J Mitochondrial pyruvate carriers are required for myocardial stress adaptation. *Nat Metab.* (2020) 2:1248–64. 10.1038/s42255-020-00288-1 33106689 PMC8015649

[44] Ho KL Zhang L Wagg C Al Batran R Gopal K Levasseur J Increased ketone body oxidation provides additional energy for the failing heart without improving cardiac efficiency. *Cardiovasc Res.* (2019) 115:1606–16. 10.1093/cvr/cvz045 30778524 PMC6704391

[45] Willis BC Salazar-Cantú A Silva-Platas C Fernández-Sada E Villegas CA Rios-Argaiz E Impaired oxidative metabolism and calcium mishandling underlie cardiac dysfunction in a rat model of post-acute isoproterenol-induced cardiomyopathy. *Am J Physiol Heart Circ Physiol.* (2015) 308:H467–77. 10.1152/ajpheart.00734.2013 25527782

[46] Nguyen TH Surikow SY Horowitz JD. Editorial commentary: Takotsubo syndrome: a key role for inflammation? *Trends Cardiovasc Med.* (2021) 31:231–2. 10.1016/j.tcm.2020.04.002 32344019

[47] Münzel T Templin C Cammann VL Hahad O. Takotsubo syndrome: impact of endothelial dysfunction and oxidative stress. *Free Radic Biol Med.* (2021) 169:216–23. 10.1016/j.freeradbiomed.2021.03.033 33864955

[48] Mao S Luo X Li Y He C Huang F Su C. Role of PI3K/AKT/mTOR pathway associated oxidative stress and cardiac dysfunction in Takotsubo syndrome. *Curr Neurovasc Res.* (2020) 17:35–43. 10.2174/1567202617666191223144715 31870264

[49] Chen B Lu Y Chen Y Cheng J. The role of Nrf2 in oxidative stress-induced endothelial injuries. *J Endocrinol.* (2015) 225:R83–99. 10.1530/JOE-14-0662 25918130

[50] Nef HM Möllmann H Troidl C Kostin S Böttger T Voss S Expression profiling of cardiac genes in Tako-Tsubo cardiomyopathy: insight into a new cardiac entity. *J Mol Cell Cardiol.* (2008) 44:395–404. 10.1016/j.yjmcc.2007.10.015 18054041

[51] Ago T Kuroda J Pain J Fu C Li H Sadoshima J. Upregulation of Nox4 by hypertrophic stimuli promotes apoptosis and mitochondrial dysfunction in cardiac myocytes. *Circ Res.* (2010) 106:1253–64. 10.1161/CIRCRESAHA.109.213116 20185797 PMC2855780

[52] Qi C Liu X Xiong T Wang D. Tempol prevents isoprenaline-induced Takotsubo syndrome via the reactive oxygen species/mitochondrial/anti-apoptosis/p38 MAPK pathway. *Eur J Pharmacol.* (2020) 886:173439. 10.1016/j.ejphar.2020.173439 32871175

[53] Qi C Shao Y Liu X Wang D Li X. The cardioprotective effects of icariin on the isoprenaline-induced Takotsubo-like rat model: involvement of reactive oxygen species and the TLR4/NF-κB signaling pathway. *Int Immunopharmacol.* (2019) 74:105733. 10.1016/j.intimp.2019.105733 31288151

[54] Akashi YJ Goldstein DS Barbaro G Ueyama T. Takotsubo cardiomyopathy: a new form of acute, reversible heart failure. *Circulation.* (2008) 118:2754–62. 10.1161/CIRCULATIONAHA.108.767012 19106400 PMC4893309

[55] Nef HM Möllmann H Troidl C Kostin S Voss S Hilpert P Abnormalities in intracellular Ca2+ regulation contribute to the pathomechanism of Tako-Tsubo cardiomyopathy. *Eur Heart J.* (2009) 30:2155–64. 10.1093/eurheartj/ehp240 19525500

[56] Kim SJ Kudej RK Yatani A Kim YK Takagi G Honda R A novel mechanism for myocardial stunning involving impaired Ca(2+) handling. *Circ Res.* (2001) 89:831–7. 10.1161/hh2101.098547 11679414

[57] Ren J Bi Y Sowers JR Hetz C Zhang Y. Endoplasmic reticulum stress and unfolded protein response in cardiovascular diseases. *Nat Rev Cardiol.* (2021) 18:499–521. 10.1038/s41569-021-00511-w 33619348

[58] Yao Y Lu Q Hu Z Yu Y Chen Q Wang QK. A non-canonical pathway regulates ER stress signaling and blocks ER stress-induced apoptosis and heart failure. *Nat Commun.* (2017) 8:133. 10.1038/s41467-017-00171-w 28743963 PMC5527107

[59] Manousek J Kala P Lokaj P Ondrus T Helanova K Miklikova M Oxidative stress in Takotsubo syndrome-is it essential for an acute attack? Indirect evidences support multisite impact including the calcium overload-energy failure hypothesis. *Front Cardiovasc Med.* (2021) 8:732708. 10.3389/fcvm.2021.732708 34738019 PMC8562109

[60] Mitchell A Marquis F. Can Takotsubo cardiomyopathy be diagnosed by autopsy? Report of a presumed case presenting as cardiac rupture. *BMC Clin Pathol.* (2017) 17:4. 10.1186/s12907-017-0045-0 28396614 PMC5382367

[61] Pascual I Abó AI Piqué M. Histological findings in Tako-Tsubo syndrome. *Rev Esp Cardiol Engl Ed.* (2015) 68:625. 10.1016/j.rec.2014.08.013 25511560

[62] Du Y Demillard LJ Ren J. Catecholamine-induced cardiotoxicity: a critical element in the pathophysiology of stroke-induced heart injury. *Life Sci.* (2021) 287:120106. 10.1016/j.lfs.2021.120106 34756930

[63] Wittstein IS Thiemann DR Lima JA Baughman KL Schulman SP Gerstenblith G Neurohumoral features of myocardial stunning due to sudden emotional stress. *N Engl J Med.* (2005) 352:539–48. 10.1056/NEJMoa043046 15703419

[64] Gupta S Goyal P Idrees S Aggarwal S Bajaj D Mattana J. Association of endocrine conditions with Takotsubo cardiomyopathy: a comprehensive review. *J Am Heart Assoc.* (2018) 7:e009003. 10.1161/JAHA.118.009003 30371307 PMC6404898

[65] Omerovic E Citro R Bossone E Redfors B Backs J Bruns B Pathophysiology of Takotsubo syndrome – A joint scientific statement from the Heart Failure Association Takotsubo syndrome study group and myocardial function working group of the European Society of Cardiology – Part 2: vascular pathophysiology, gender and sex hormones, genetics, chronic cardiovascular problems and clinical implications. *Eur J Heart Fail.* (2022) 24:274–86. 10.1002/ejhf.2368 34655287

[66] Hiestand T Hänggi J Klein C Topka MS Jaguszewski M Ghadri JR Takotsubo syndrome associated with structural brain alterations of the limbic system. *J Am Coll Cardiol.* (2018) 71:809–11. 10.1016/j.jacc.2017.12.022 29447745

[67] Suzuki H Matsumoto Y Kaneta T Sugimura K Takahashi J Fukumoto Y. Evidence for brain activation in patients with Takotsubo cardiomyopathy. *Circ J.* (2014) 78:256–8. 10.1253/circj.cj-13-1276 24284957

[68] Khan H Rudd A Gamble DT Mezincescu AM Cheyne L Horgan G Renin-angiotensin and endothelin systems in patients post-Takotsubo cardiomyopathy. *J Am Heart Assoc.* (2022) 11:e025989. 10.1161/JAHA.122.025989 35861811 PMC9707811

[69] Vaccaro A Despas F Delmas C Lairez O Lambert E Lambert G Direct evidences for sympathetic hyperactivity and baroreflex impairment in Tako Tsubo cardiopathy. *PLoS One.* (2014) 9:e93278. 10.1371/journal.pone.0093278 24667435 PMC3965544

[70] Shahim B Faxén UL Stern R Freyschuss A. Cardiogenic shock triggered by phaeochromocytoma crisis after an oral glucose tolerance test: a case report. *Eur Heart J Case Rep.* (2019) 3:1–7. 10.1093/ehjcr/ytz177 31911981 PMC6939798

[71] Ribas V Nguyen MT Henstridge DC Nguyen AK Beaven SW Watt MJ. Impaired oxidative metabolism and inflammation are associated with insulin resistance in ERalpha-deficient mice. *Am J Physiol Endocrinol Metab.* (2010) 298:E304–19. 10.1152/ajpendo.00504.2009 19920214 PMC2822483

[72] Barbiero S Aimo A Castiglione V Giannoni A Vergaro G Passino C Healthy hearts at hectic pace: from daily life stress to abnormal cardiomyocyte function and arrhythmias. *Eur J Prev Cardiol.* (2018) 25:1419–30. 10.1177/2047487318790614 30052067

[73] Kofron CM Mende U. In vitro models of the cardiac microenvironment to study myocyte and non-myocyte crosstalk: bioinspired approaches beyond the polystyrene dish. *J Physiol.* (2017) 595:3891–905. 10.1113/JP273100 28116799 PMC5471366

[74] Dawson DK Neil CJ Henning A Cameron D Jagpal B Bruce M TakoTsubo cardiomyopathy: a heart stressed out of energy? *JACC Cardiovasc Imaging.* (2015) 8:985–7. 10.1016/j.jcmg.2014.10.004 25499134

[75] Schwarz K Ahearn T Srinivasan J Neil CJ Scally C Rudd A Alterations in cardiac deformation, timing of contraction and relaxation, and early myocardial fibrosis accompany the apparent recovery of acute stress-induced (takotsubo) cardiomyopathy: an end to the concept of transience. *J Am Soc Echocardiogr.* (2017) 30:745–55. 10.1016/j.echo.2017.03.016 28599831

[76] Napp LC. The risk of takotsubo syndrome: seeing the light. *JACC Heart Fail.* (2019) 7:155–7. 10.1016/j.jchf.2018.11.012 30611721

[77] Ghadri JR Cammann VL Jurisic S Seifert B Napp LC Diekmann J A novel clinical score (intertak diagnostic score) to differentiate takotsubo syndrome from acute coronary syndrome: results from the international takotsubo registry. *Eur J Heart Fail.* (2017) 19:1036–42. 10.1002/ejhf.683 27928880

[78] Sato A Aonuma K Nozato T Sekiguchi Y Okazaki O Kubota K Stunned myocardium in transient left ventricular apical ballooning: a serial study of dual I-123 BMIPP and Tl-201 SPECT. *J Nucl Cardiol.* (2008) 15:671–9. 10.1016/j.nuclcard.2008.03.010 18761270

[79] Owa M Aizawa K Urasawa N Ichinose H Yamamoto K Karasawa K Emotional stress-induced ‘ampulla cardiomyopathy’: discrepancy between the metabolic and sympathetic innervation imaging performed during the recovery course. *Jpn Circ J.* (2001) 65:349–52. 10.1253/jcj.65.349 11316138

[80] Y-Hassan S Tornvall P. Reply to: metoprolol, or propranolol, or carvedilol, or labetalol, for patients with takotsubo syndrome? *Clin Auton Res.* (2018) 28:133–4. 10.1007/s10286-017-0483-x 29181766

[81] Y-Hassan S Tornvall P. Epidemiology, pathogenesis, and management of takotsubo syndrome. *Clin Auton Res.* (2018) 28:53–65. 10.1007/s10286-017-0465-z 28917022 PMC5805795

[82] Isogai T Matsui H Tanaka H Fushimi K Yasunaga H. Early β-blocker use and in-hospital mortality in patients with takotsubo cardiomyopathy. *Heart.* (2016) 102:1029–35. 10.1136/heartjnl-2015-308712 26879240

[83] Templin C Ghadri JR Diekmann J Napp LC Bataiosu DR Jaguszewski M Clinical features and outcomes of takotsubo (stress) cardiomyopathy. *N Engl J Med.* (2015) 373:929–38. 10.1056/NEJMoa1406761 26332547

[84] Ueyama T. Emotional stress-induced tako-tsubo cardiomyopathy: animal model and molecular mechanism. *Ann N Y Acad Sci.* (2004) 1018:437–44. 10.1196/annals.1296.054 15240400

[85] Beanlands RS Nahmias C Gordon E Coates G deKemp R Firnau G The effects of beta-blockade on oxidative metabolism and the metabolic cost of ventricular work in patients with left ventricular dysfunction: a double-blind, placebo-controlled, positron-emission tomography study. *Circulation.* (2000) 102:2070–5. 10.1161/01.cir.102.17.2070 11044422

[86] Zhang Z Jin S Teng X Duan X Chen Y Wu Y. Hydrogen sulfide attenuates cardiac injury in takotsubo cardiomyopathy by alleviating oxidative stress. *Nitric Oxide.* (2017) 67:10–25. 10.1016/j.niox.2017.04.010 28450188

[87] Ciutac AM Dawson D. The role of inflammation in stress cardiomyopathy. *Trends Cardiovasc Med.* (2021) 31:225–30. 10.1016/j.tcm.2020.03.005 32276825

[88] Marfella R Barbieri M Sardu C Rizzo MR Siniscalchi M Paolisso P Effects of α-lipoic acid therapy on sympathetic heart innervation in patients with previous experience of transient takotsubo cardiomyopathy. *J Cardiol.* (2016) 67:153–61. 10.1016/j.jjcc.2015.07.012 26347218

[89] Santoro F Mallardi A Leopizzi A Vitale E Rawish E Stiermaier T Current knowledge and future challenges in Takotsubo syndrome: part 2-treatment and prognosis. *J Clin Med.* (2021) 10:468. 10.3390/jcm10030468 33530545 PMC7866173

